# Supplementary material for: Optimal control prevents itself from eradicating stochastic disease epidemics
Source: PLoS Comput Biol. 2025 Feb 18;21(2):e1012781. doi: 10.1371/journal.pcbi.1012781 (PMC11844887; doi:10.1371/journal.pcbi.1012781)
Supplement: S1 Appendix — Plots and descriptions of the trajectories of infected hosts and applied control for a subset of the subpopulations in the 4x4 intermediate system. (PDF) [file pcbi.1012781.s001.pdf]

Optimal Control Prevents Itself from Eradicating Stochastic Disease Epidemics: Supplement S1

Rachel Russell, Nik J. Cunniffe

In order to provide some further intuition on the differences in behaviour for the epidemic system with MPC based control and with priority based control, this supplement contains plots showing individual trajectories of the number of infected hosts and the applied control for a subset of the subpopulations in the 4x4 intermediate system. For each set of plots, the different rows of plots are different iterations of the random simulation (different random seeds) and the columns correspond to different subpopulations in the system. Note that only a subset of the nodes is shown corresponding to one “edge” of the 4x4 square.

Using the MPC control (Figure 1), the blue line showing the rate of culling tracks the red line of infected hosts relatively closely. We can also see where the MPC is predicting that the infected population will increase during the control timestep e.g., in the first cycle for subpopulation 1 and in cycles 4 to 10 for subpopulation 5 seed 2 (highlighted with green circles). In comparison, we can see the overspend behaviour in the prioritised control (Figure 2). At the points where the infected population is driven to zero, we often see large overshoots of control (again highlighted with green circles). We can also see switching behaviour where the focus of control moves between the nodes from one timestep to the next.

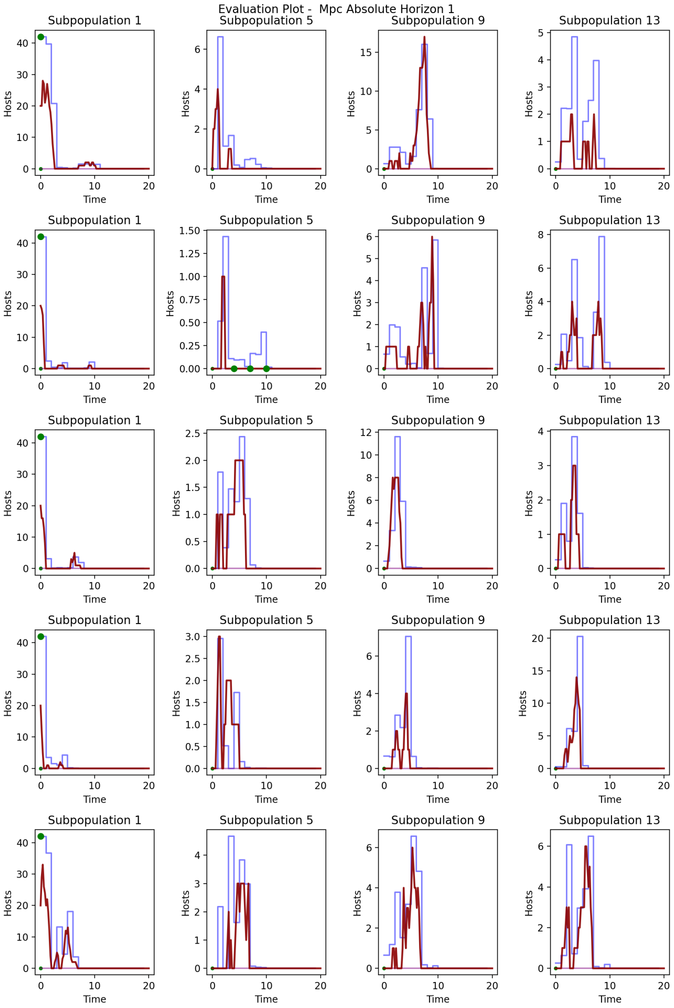

Figure 1: Example trajectories for the 4x4 intermediate system with MPC control. The red line is the number of infected hosts in the subpopulation, the blue line is the rate of culling applied to the subpopulation in hosts per unit time. Notable behaviour discussed in the main text is highlighted with green circles.

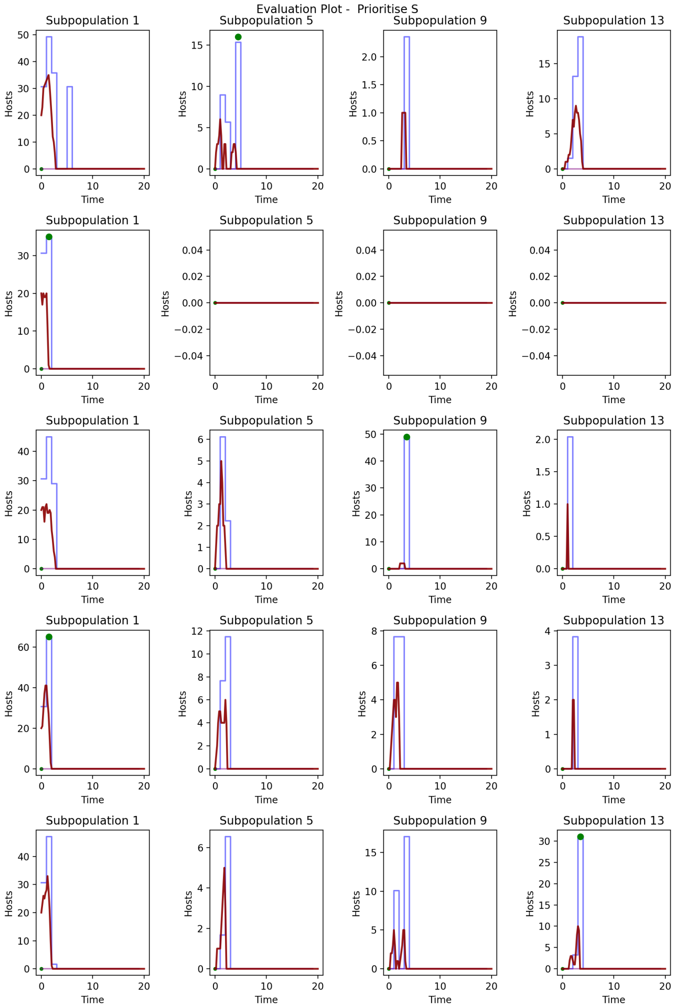

Figure 2: Example trajectories for the 4x4 intermediate system controlled with the prioritise S heuristic. As per the Figure 1, the red line is the number of infected hosts in the subpopulation, the blue line is the rate of culling applied to the subpopulation in hosts per unit time. Notable behaviour discussed in the main text is highlighted with green circles.
