# Supplementary material for: Optimal control prevents itself from eradicating stochastic disease epidemics
Source: PLoS Comput Biol. 2025 Feb 18;21(2):e1012781. doi: 10.1371/journal.pcbi.1012781 (PMC11844887; doi:10.1371/journal.pcbi.1012781)
Supplement: Attachment — Submitted filename: PLOS_Comp_Bio_Review-2.pdf [file pcbi.1012781.s002.pdf]

Review: Optimal Control Prevents Itself from Eradicating Stochastic Disease Epidemics

PLOS Computational Biology Anonymous Reviewer

June 2024

1 Summary

The authors study the performance of deterministic optimal control, model predictive control, and several simple heuristic policies at eradicating *stochastic* epidemics. The focus on eradication draws focus to regimes in which the number of individuals is low, increasing the estimation error of mean field approximations and deterministic models which rely on the Central Limit Theorem for convergence as the population approaches infinity.

In this setting, the authors experiments show that optimal control strategies do not eradicate the epidemic when the true state diverges from that predicted by the deterministic system dynamics. In particular, the deterministic model may underestimate the future infected population. Since control is constrained by the deterministic dynamics, the resulting optimal controller does not fully eradicate the disease. The authors then consider an MPC variant where the control is recalculated periodically with updated state information. While this improves state estimation and performance, the main problem remains – when the state estimate diverges from reality, the controller may incur significant suboptimality, potentially even choosing to not eradicate the disease.

Overall, the work is clearly written. The problem of controlling epidemics and computational methodologies is of import.

2 Major Suggestions and Comments

1. It is unclear to me whether the eradication scenario is a particularly important one for considering optimization. Generally, resource allocation is most important when the control is heavily constrained and lower than the target population. In the eradication scenario, control is not under such stringent constraint relative to the population. It seems that in this situation.

2. It would also seem that the divergence between true state and the estimate would decrease if one considers a finer discretization in time. Maybe this

would result in computational intractability (if the state space becomes too large), but the authors should address this either way.

3. Te authors do not address methods in robust optimal control, stochastic optimal control, or state estimation which could tackle the problem. I have included links to a few relevant works below and many more exist.
   - https://www.medrxiv.org/content/10.1101/2023.12.10.23299100v1.article-info
   - https://www.nature.com/articles/s41598-020-67459-8
   - https://www.sciencedirect.com/science/article/pii/S0005109811000410
4. The optimal control formulation penalizes the controller for excessive culling or thinning because its objective is to maximize the susceptible population. In reality, if eradication is being strongly considered, it is reasonable to assume that the controller may be liberal in the application of control, perhaps even culling or thinning susceptible individuals. Wouldn't this counteract the potential for underestimating the infected population?

3 Other Feedback

1. It would be useful to see the actual policies found by the optimal control and MPC methods. How do they compare qualitatively to the heuristic baselines?
2. With the focus of the paper, it seems that more attention should be paid to problems related to noisy or partial state knowledge. This seems to be the crux of the problem the authors broach.
3. It would useful to discuss more substantively how your results can be used by practitioners working to control an epidemic or determine strategy to do so.
