# Supplementary material for: Optimal control prevents itself from eradicating stochastic disease epidemics
Source: PLoS Comput Biol. 2025 Feb 18;21(2):e1012781. doi: 10.1371/journal.pcbi.1012781 (PMC11844887; doi:10.1371/journal.pcbi.1012781)
Supplement: Attachment — Submitted filename: OCvsRiskBasedCoverLetterRevisions.pdf [file pcbi.1012781.s003.pdf]

Rachel Russell
Department of Plant Sciences
University of Cambridge
Downing Street
Cambridge, CB2 3EA

UNIVERSITY OF CAMBRIDGE
Department of Plant Sciences

5th November 2024

Optimal control prevents itself from eradicating stochastic disease epidemics

Rachel Russell and Nik J. Cunniffe

Dear Editor,

Thank you for considering our manuscript for publication in *PLOS Computational Biology* and for your patience in allowing us a slightly longer window for resubmission. As per previous correspondence, I have been on an industrial placement for the past three months and responding to this review has been my first priority on my return. Thank you also to the reviewers for providing valuable feedback on our submission. We have made significant updates to the content of the paper and we hope that we have properly understood and addressed the constructive criticism that was provided. Below this letter we have provided a detailed list of our responses to the reviewers' comments and a description of the changes made in the manuscript to address each point. As requested in the decision email, I have provided a marked up copy of the manuscript showing the changes. The changes triggered by Reviewer 1's comments are marked "r1" and are in blue text. The changes from Reviewer 2's comments are marked "r2" and are in purple. The line numbers used in the review responses are from the updated manuscript with additions and deletions highlighted.

We hope that these changes will address the reviewers' comments but also welcome further feedback as required.

Kind regards,

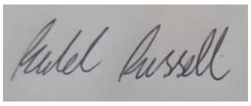

Rachel Russell (corresponding author on behalf of both co-authors)

Response to Reviewer 1

"1. I suggest rewriting the author summary to make it more clear. In Author summary:
- Optimisations –spelling check
- Allocating limited resources to reduce the impact of an epidemic is important across human, animal and plant health. – not clear
- For example, deciding which infected plants to remove first to reduce ecosystem or agricultural losses from an invasive plant disease [is important/critical?] – incomplete sentence.
- A common approach to these optimisations is to model the epidemic as a totally predictable (deterministic) process and modern algorithms allow these optimisations to scale to large, real-world problems (e.g. allocating resources across a landscape). Which optimizations are being referred here?"

> I've reworded the author summary to clarify the points raised, apart from the first, since I believe that optimise / optimisations are valid (British) English spellings and have been used in PLOS journals in the past (e.g. https://journals.plos.org/plosbiology/article%3Fid%3D10.1371/journal.pbio.3000863). In the interests of consistency, I've tried to use British English throughout and I don't believe that American English is

specified in the submission guidelines. Obviously, I'm happy to change to match house style if American spelling is more appropriate.

Action: Changed text in first 6 lines of author summary.

"2. Introduction - Management aims vary dependent on the scenario and pathosystem in question (see [6] for a review of the equivalent problem in the context of established invasive species). Which management?"

This has been reworded in the new version.
Action: Changed text on line 12.

3. There are Hyphens (-) inside a few of the sentences throughout.

Thanks - I always struggle to spot these!
Action: I have done a find and replace for " - " to remove them. Note that these are not included in the tracked changes).

4. It is mentioned that the plausible assumptions is that human decision making happens at discrete intervals and that there may be some delay between the decision making and the action being carried out. In that case, is this delay incorporated in the ODE's or anywhere else? If not, give reasons

The idea here was that modelling the control with a constant stochastic rate within each timestep would mimic the delay between decision making and action being carried out. This has been reworded in the text to try to be clearer.
Action: Changed text on lines 127-132

5. FIGURES placements need to be fixed. Some of them overflowing from the page and not labeled

I'm sorry but I'm afraid I can't see this problem - the version I see when I download the submission has the figures provided separately and doesn't show the layout of the figures at all. I will happily work with the editorial team to fix any figure formatting issues if the other changes are acceptable, but I suspect this is a function of the particular version of the manuscript supplied for review.

6. Consistency in references to citations

I'm afraid I'm not sure what this is referring to. In the version available to me, the numbering style used for references appears consistent. As per the previous comment, I'm very happy to work to correct any issues if they are present in the final version.

Response to reviewer 2

Q1: "It is unclear to me whether the eradication scenario is a particularly important one for considering optimization. Generally, resource allocation is most important when the control is heavily constrained and lower than the target population. In the eradication scenario, control is not under such stringent constraint relative to the population. It seems that in this situation."

For the simple cases illustrated in the some of the examples, you are correct. It doesn't make sense to try to optimise resource allocation if the system is very small relative to the amount of resource available. However, the examples shown are a way of demonstrating issues that could also happen in more general scenarios - e.g. local eradication. In addition, resources are still not infinite even in cases where eradication is feasible. There is still prioritisation to be done between the different subpopulations unless you have enough resource to eradicate everything immediately. Immediate eradication was feasible in the simpler cases shown but this was only to illustrate the mechanics of the underestimates which occur when using optimal control. The case where eradication is feasible but requires prioritisation is particularly shown in the intermediate cost scenarios. To address this feedback, I have added some extra description around the intermediate cost scenarios to try to make this clearer. It is also perhaps important to note that there are high profile examples in which eradication or an attempted eradication has continued for a lengthy period of time – examples from plant diseases include citrus canker in Florida (actively "under eradication" from 1998-

2006 before it was determined eradication had failed) Xylella fastidiosa on almond in Spain (the eradication programme that started in 2017 is still ongoing) - and in these cases in which eradication was far from immediate it is clear that resource limitation would have been an important factor.

Action: Added text on lengthy eradications to Introduction (lines 21-24), extended text on intermediate cost scenarios and moved to make more prominent (lines 347-359, lines 371-376)

Q2: "It would also seem that the divergence between true state and the estimate would decrease if one considers a finer discretization in time. Maybe this would result in computational intractability (if the state space becomes too large), but the authors should address this either way."

It is true that the divergence would be smaller if the time discretisation of control was smaller relative to the rate of spread of the epidemic. However, we argue that this time step is governed by real world constraints rather than computational constraints. There are two types of discretisation in the optimisation we consider, and we deliberately made them separate because they are governed by different factors. The first is the discretisation of the optimal control calculations (performed at finer resolution). I would not expect reducing this timestep to significantly affect the issues that are highlighted. This is governed by a trade off between computational expense and the ability of the optimisation algorithm to converge (if you make it too coarse relative to the speed of dynamics, the ipopt algorithm fails to converge; if you make it too fine, the computation takes too long). The second discretisation interval is the discretisation of the control intervals (coarser resolution). We deliberately made this separate to the optimal control timestep because this is not governed by computational considerations but by logistical constraints (how often surveys can be carried out and/or how often an alternative policy for control can be introduced). You are right that finer discretisation of this interval would make the divergence smaller; the problem is that in the real world the finest realistic discretisation is set by practical/logistic considerations outside of our control. The interesting result here is that the simpler algorithms can be effective with the lower frequency surveys. I have added some description in the text to make this point more clearly.

Action: Added text – lines 507-514

Q3: "Te authors do not address methods in robust optimal control, stochastic optimal control, or state estimation which could tackle the problem. I have included links to a few relevant works below and many more exist.

• https://www.medrxiv.org/content/10.1101/2023.12.10.23299100v1.article- info

• https://www.nature.com/articles/s41598-020-67459-8

• https://www.sciencedirect.com/science/article/pii/S0005109811000410 "

Thank you - this is good feedback about how we have presented the work. The main point we had not brought out in the text is that, although there are several common frameworks for addressing uncertainty and noise in optimal control, there isn't an existing framework that applies in a principled and scalable way to stochastic epidemic systems when pathogen populations are close to eradication. The standard frameworks assume additive noise on the system state or inaccuracy in the parameterisations and there is no mechanism to sensibly map between these types of models and a stochastic process in the area of the state space we are interested in. I suppose one could also challenge our work by saying we should change the epidemic models to match the assumptions of the control more closely - assuming a stochastic process is just as much of an assumption as assuming additive noise. However, we argue that using stochastic processes is both more principled and more common within epidemiology. This is most clearly set out in Keeling and Rohani – a standard textbook in mathematical epidemiology - Chapter 6.2.4.2 at the end of their description of the noise-based models: "These stochastic equations suffer from one major drawback: They do not incorporate the discrete, individual nature of populations and are therefore not a suitable model when population levels (e.g. the number of infectious individuals) are small." However, we could also make

this case by considering the vastly larger number of papers focusing on representations of real-world epidemics using individual based stochastic processes, rather than via additive noise.

Action: Extended and reworded text in Discussion section (lines 446-471)

Taking each of the proposed methods and links in turn:

a. robust optimal control

Robust optimal control aims to make the control robust against imperfections in model parameterisations and system state knowledge. The methods optimise the worst case trajectory and/or ensure that worst case trajectories do not go outside of the system constraints. There are two main mismatches between our problem and the formulation for robust optimal control. Firstly and most importantly, we are not concerned if the trajectories violate the system constraints in the deterministic system. The main constraint is that the number of hosts in each compartment should remain above zero and this is required to keep the model in a realistic region of state space rather than representing a real world constraint. In some respects, this inability to violate the zero infected constraint in the deterministic world could be seen as the root of the issues we have seen. Additionally, the aim of robust optimal control is to guard against imperfections in knowledge about parameters and we assume that we have perfect knowledge of the parameters and intermittent perfect knowledge of the system state. I have updated the text around robust optimal control in the discussion section to explain this in more depth (from line 450).

b. Stochastic optimal control

Analytical solutions to stochastic optimal control problems scale poorly with system size and so would be computationally intractable for realistic scales of systems. Discussion of this approach was already included as part of the Introduction text on lines 38-48 and in the Discussion on lines 379-391. Modern approaches to these systems focus on approximating solutions with e.g. deep reinforcement learning. These methods are outside the scope of what we are presenting in this paper. However, one reading of this work is that it justifies further exploration of approximate methods like RL and it clarifies one type of problem where they could usefully be considered.

Action: Added text to clarify terminology (line 385)

c. State estimation

There are several ways we could consider using state estimation in this context. However, I don't think any of them provide a solution for the case we have presented. In the example provided (link f), state estimation is used to infer hidden states. This is not relevant here as we are assuming that the state of each host is visible at each timestep. We could also look at state estimation in the sense of estimating how the states will evolve through the course of each timestep (between observations). This happens as a matter of course within the optimal control algorithm. However, this extrapolation assumes a deterministic evolution of the state. In order for state estimation to be beneficial in the case we present, it would require a mechanism to calculate and account for the expected levels of divergence through the time step into the optimal control algorithm. I am not aware of any literature covering this type of approach although it is a sensible area for future investigation. I have added this to the manuscript as an area for future consideration (line 462-464).

d. https://www.medrxiv.org/content/10.1101/2023.12.10.23299100v1.article-info

Apologies if I have misunderstood your intent but this paper does not seem to use any of the methodologies mentioned in the comment and so the relevance of the paper to the feedback is not entirely clear. This paper uses deterministic optimal control to determine the best vaccination strategy for COVID-19 in a heterogenous population structure by optimising a system of 30 ODEs (10 disease state x 3 contact level model). Footnote 2 in this paper describes very well the key assumptions used in this paper and in the majority of papers in this field to justify the use of deterministic ODEs for a stochastic system. Our work highlights an important area of the state space where these assumptions are not valid.

e. https://www.nature.com/articles/s41598-020-67459-8

This paper is solving a different problem to the problem we are presenting in our work and it is working in an area of the epidemic state space where stochasticity can safely be ignored. In the paper, the optimisation is carried out on a deterministic system and the stochasticity is not modelled other than as a measurement error (modelled as additive noise). Linking back to the methods in your comment, the authors do use state estimation but this is used to account for unmeasurable states in the covid model. This is not relevant for our work because we already assume perfect knowledge of the system state at each time step. The issues we are highlighting occur even without any additional inaccuracies introduced by this kind of state estimation.

f. https://www.sciencedirect.com/science/article/pii/S0005109811000410

This paper describes a refinement of the "Linear Quadratic Gaussian regulator (LQG, Åström, 1970)". In order to use this type of control methodology, the system needs to be framed as a linear system with additive Gaussian noise. As discussed at the start of our response, our work is looking at a different type of system where the stochastic behaviour cannot be adequately represented by the addition of noise.

"Q4: The optimal control formulation penalizes the controller for excessive culling or thinning because its objective is to maximize the susceptible population. In reality, if eradication is being strongly considered, it is reasonable to assume that the controller may be liberal in the application of control, perhaps even culling or thinning susceptible individuals. Wouldn't this counteract the potential for underestimating the infected population?"

Changing the formulation of the reward would not change or counteract the controller's potential for underestimating the infected population. In the examples shown, the controller is using the maximum level of control available to it to remove all the infected hosts. If it increases the level of control any further, the internal deterministic model of the epidemic system will have a negative value of infected hosts at the end of the timestep. The optimal control has constraints preventing it from entering this area of the state space. These constraints cannot be removed because without them the model can be driven into nonsensical states (which the controller will prefer because they generate high rewards – a negative infected host is effectively a host which heals other hosts). I believe this was already included in the manuscript in the "Potential challenges" section (line 519: "Similarly, it may be possible and pragmatic to bias the optimal control towards scenarios where local or global eradication is likely by using a different reward formulation..."). However, we can include further discussion of this point, should the Editor request.

Other feedback:

Q1: "It would be useful to see the actual policies found by the optimal control and MPC methods. How do they compare qualitatively to the heuristic baselines?"

Action: Added as supplementary material.

Q2: "With the focus of the paper, it seems that more attention should be paid to problems related to noisy or partial state knowledge. This seems to be the crux of the problem the authors broach."

I believe this has been addressed in my response to Q3c.

Q3: "It would useful to discuss more substantively how your results can be used by practitioners working to control an epidemic or determine strategy to do so."

I have added some extra thoughts on this in the discussion section.

Action: Added text - line 584-594.

Thanks again! RR.
